# Thymus and Mediastinal Node Involvement in Childhood Langerhans Cell Histiocytosis: Long-Term Follow-Up From the French National Cohort

**DOI:** 10.1002/pbc.24603

**Published:** 2013-06-29

**Authors:** Stephane Ducassou, Fanny Seyrig, Caroline Thomas, Anne Lambilliotte, Perrine Marec-Berard, Claire Berger, Genevieve Plat, Laurence Brugiere, Marie Ouache, Mohamed Barkaoui, Corinne Armari-Alla, Patrick Lutz, Guy Leverger, Xavier Rialland, Ludovic Mansuy, Helene Pacquement, Eric Jeziorski, Virginie Gandemer, François Chalard, Jean François Chateil, Abdellatif Tazi, Jean François Emile, Jean Donadieu, the Investigators of the French LCH Study Group

**Affiliations:** 1 Service D'hémato Oncologie Pédiatrique, CHU de StrasbourgStrasbourg, France; 2 Service D'hémato Oncologie Pédiatrique, CHU de BordeauxBordeaux, France; 3 Service D'hémato Oncologie Pédiatrique, CHU de NantesNantes, France; 4 Service D'hématologie Oncologie Pédiatrique, Hôpital Jeanne de FlandresLille, France; 5 Institut D'hémato Oncologie Pédiatrique, Hospice civil de LyonLyon, France; 6 Service D'hémato Oncologie Pédiatrique, CHU de Saint EtienneFrance; 7 Service D'hémato Oncologie Pédiatrique Hopital Purpan, CHU de ToulouseFrance; 8 Service D'oncologie Pédiatrique, Institut Gustave RoussyVillejuif, France; 9 Service D'hématologie Pédiatrique, Hopital Robert DébréParis, France; 10 Service D'hémato Oncologie Pédiatrique, Centre de référence des histiocytoses, registre des histiocytoses, APHP Hôpital A. TrousseauParis, France; 11 Service de Pédiatrie, Unité D'hémato Oncologie Pédiatrique, CHU MichallonGrenoble, France; 12 Service de Pédiatrie, Unité d'hémato Oncologie Pédiatrique, CHU d'AngersAngers, France; 13 Service de Médecine Infantile II, CHU de Brabois, NancyFrance; 14 Service de Pédiatrie, Institut CurieParis, France; 15 Service de Médecine Infantile, Hopital Arnaud de Villeneuve, CHU de MontpellierMontpellier, France; 16 Service D'hémato Oncologie Pédiatrique, Hopital Sud CHURennes, France; 17 Service de Radiologie APHP, Hopital TrousseauParis, France; 18 Service Radiologie Pédiatrique, CHU BordeauxFrance; 19 Service de Pneumologie, Centre de référence des histiocytoses, APHP Hôpital Saint LouisParis, France; 20 Laboratoire D'anatomie et Cytologie Pathologique, APHP Hopital A ParéBoulognes, France

**Keywords:** Langerhans cell histiocytosis, mediastinal nodes, survey, thymus

## Abstract

**Background:**

Mediastinal involvement (MI) in Langerhans cell histiocytosis (LCH) has been rarely reported. Here, we describe the clinical, radiological, and biological presentation, and the outcome of childhood LCH with MI.

**Method:**

From the French LCH register, which includes 1,423 patients aged less than 18 years, we retrieved the medical charts of patients with mediastinal enlargement detected on chest X-rays.

**Results:**

Thirty-seven patients were retrieved, including 18 males; median age of diagnosis was 0.7 years, and median follow-up time was 6.2 years. The prevalence of MI varied with the age at diagnosis, ranging from 7% below 1 year old to less than 1% at >5 years. Thirteen cases (35%) were diagnosed because of MI-related symptoms, including respiratory distress (N = 4), superior venous cava syndrome (N = 2), and/or cough and polypnea (N = 10). CT scans performed in 32 cases at diagnosis showed tracheal compression (N = 5), cava thrombosis (N = 2), and/or calcification (N = 16). All patients presented multi-system disease at LCH diagnosis, and 35/37 were initially treated with vinblastine and corticosteroids. Death occurred in five cases, due to MI (N = 1) or hematological refractory involvement (N = 4). The overall 5-year survival was 87.1%, and immunodeficiency was not detected as a sequel.

**Conclusions:**

MI in LCH mainly occurs in young children, and diagnosis was based on CT showing thymus enlargement and calcifications. Pediatr Blood Cancer 2013;60:1759–1765. © 2013 Wiley Periodicals, Inc.

## INTRODUCTION

Langerhans cell histiocytosis (LCH) is a disease characterized by accumulation of Langerhans cells in various tissues or organs; about half of cases involve mutation of the B-raf oncogene 1. LCH clinical presentation and outcome are heterogeneous, ranging from an isolated spontaneously remitting lesion to a multi-system disease including sometimes life-threatening organ dysfunction 2. Rarely, thymus and mediastinal lymph node involvement is observed; an exhaustive literature search covering 50 years identified no more than 50 cases reported to date (Table I). In the present study, we use a clinical national registry that was established in 1993 to systematically review all patients with LCH who presented thymic or mediastinal involvement (MI). Using this information, we describe the natural history of this rare involvement in LCH.

**Table I:** Literature Review of Pediatric and Adult Cases (1963–2011)

Publication year [Ref.]	No. of cases	Age (years)	Clinical symptoms	Follow-up/outcome
Pediatric cases of Langerhans cell histiocytosis with thymic involvement				
1963 5	1	12	Arthralgia, headache, anorexia	2 years/alive
1982 6	1	1.25	Cough/weakness/anorexia	6 months/alive
1985 7	4	5, 8, 5, and 0.2	No symptoms (2)/low back pain (1)/respiratory distress (1)	4.4 years/alive
1986 8	1	0.66	No respiratory symptoms	Alive after one relapse
1985 9	1	1.5	No respiratory symptoms, soft swelling of the forehead, lung X-rays abnormal	MD
1987 10	4	2, 1, 3, and 1.3	Shortness of breath (2)/no respiratory signs (2)	3.5, 4, or 20 years/alive
1987 11	2	0.3	Lymph node enlargement, slight dyspnea and perioral subcyanosis (1)/polypnea, subcyanosis, and hepatosplenomegaly	2 years and 3 months/alive
1987 12	1	0.4	Jaundice, MOF, thymic involvement	11 months/dead
1991 13	2	0.4	Tachypnea (1) or general signs (1)	5 years/alive
1993 14	1	1.5	Superior vena cava syndrome	1 years/alive
1993 15	1	0.05	Respiratory distress	No relapse
1997 16	1	0.6	Pulmonary symptoms, skin, liver, lymph nodes	10 years/alive
1999 17	4	0.7	MD	Alive
1999 18	5	1.9 (median value)	MD	Alive
1999 19	1	2	No respiratory symptoms/abdominal pain	MD
2000 20	1	1.6	6 months of progressive dyspnea	MD
2002 21	1	0.3	Feeding difficulties, poor weight gain, SS	3 years/alive
2006 22	3	0.8	MS disease	6 months/dead, 2 years/dead, 8 years/alive
2007 23	1	MD	MD	MD
2008 24	1	1.5	Breathing difficulty, fever	MD
2008 25	2	0.4 (for the two patients)	MD	MD
2008 26	1	12	Dyspnea, chest pain, weight loss, MS hystiocytosis	1 years/alive with residual mass
2009 27	1	0.2	Fever, cough, swelling in left supraclavicular area	2 years/alive
2009 28	1	0.8	SUDI, MS histiocytosis	Dead
2010 29	1	0.25	Respiratory distress	MD
Thymic involvement discovered on incidental thymectomy or post-mortem without any clinical or radiological signs				
2003 30	1	0.9	Incidental thymectomy	19 months after surgery/alive
1986 31	1	36	Myasthenia gravis, few LCH cluster on thymectomy	MD
1997 32	1	30	Myasthenia gravis, few LCH cluster on thymectomy	
1989 33	1	21	Myasthenia gravis	Alive
2003 34	1	49	3 years after a soft tissue sarcoma: sub-sternal chest pain, leading to the discover of an anterior mediastinal mass	Alive
Adult cases of Langherans cell histiocytosis with thymic involvement				
2000 35	1	58	Acute severe right-sided chest pain	Died 2 weeks after surgery
2008 36	1	43	MD	Alive

MD, missing data; MS, multi-system; SUDI, sudden infant death.

## METHODS

### Patients

LCH diagnosis was based on the Histiocyte Society criteria 3, including morphologic features of histiocytic granuloma and additional criteria, such as CD1a expression. The French Langerhans' Cell Histiocytosis Study Group database was initially created for a retrospective study of LCH from 1983 to 1993 2; since then, enrollment has been prospective. From April 1996 to November 2001, eligible patients have been included in Histiocyte Society clinical trials (the LCH II and LCH III studies). Since 2008, this collection of data has been recognized as a national register by the French health authorities and has been verified against multiple separate sources 4. The database has been allowed by the French authorities for health data collection (Comité Consultatif pour le Traitement de l'Information en Matière de Recherche pour la Santé [CCTIRS] and Commission Nationale Informatique et Liberté [CNIL]), and patients or parents had to provide informed consent to be included in the registry. Data monitoring was performed by a clinical research associate who visited each center and reviewed medical and radiological charts. All patients with mediastinal node and/or thymus involvement were screened by chest X-rays, and thoracic computed tomography (CT) was performed in 32 cases. Following standard radiological practice, MI was diagnosed based on mediastinal area enlargement on chest X-rays 37. Since anatomical thymus and mediastinal nodes enlargement may be associated and difficult to differentiate, we reported them together. When available, CT scans provided further information about mediastinal node size and aspects of the thymus, including thymic shape (nodular or lobulated), intrathymic calcifications, and cavitations. For the present study, we systematically reviewed the medical charts, and the radiological and pathological reports. Disease activity scores were based on a previous publication 38.

### Statistical Methods

Stata® version 10 software was used for all statistical analyses. Median values and lower and upper interquartile values depict the distribution of quantitative variables. To calculate the reactivation rate, we considered the period from the MI to the onset of any new involvement or to the last visit if no additional involvement occurred. The Kaplan–Meier method was used to estimate survival. The cut-off date for this analysis was September 30, 2012.

## RESULTS

### Demography and Clinical Presentation

Of 1,423 patients enrolled in the French LCH register, 37 (2.6%) were diagnosed as having mediastinal enlargement on chest X-rays and were eligible for this study. Table II presents the demographics, organ involvement, and immunological features of these patients. They included 18 males (48%), and the median age at diagnosis of LCH was 0.7 years (IQR, 0.5–1.7 years). In four cases, the diagnosis of MI was made after the age of 10 years old. The median follow-up was 6.2 years (IQR, 1.5–11.7 years).

**Table II:** Demographic Description, Organ Involvement, and Immunological Features of the 37 Reported Patients

Characteristic	At LCH thymus diagnostic, N (%)	Maximal extent of the disease, N (%)
Age (years), median [IQR]	0.7 [0.6–1.7]	
Follow-up, median [IQR] in years	6.2 years [1.5–11.7]	
Sex (female/male)	19/18	
Disease activity score		
0–2	2 (5.4)	
3–6	24 (65)	
>6	11 (30)	
Organ involvement		
Risk organ		
Hematopoietic system	17 (46)	18 (48)
Liver	16 (43)	17 (46)
Spleen	10 (27)	12 (33)
No risk organ		
Lung	15 (40)	18 (48)
Bone	21 (56)	24 (64)
General symptoms (fever/loss of weight or failure to thrive)	23 (64)	24 (65)
Hypophysis	3 (8)	10 (27)
ENT including external auditory tract and temporal bone	13 (35)	18 (43)
Central nervous system	1 (2.7)	4 (10)
Immunological disorder (% among informative patients)		
Lymphopenia	6 (19)	
Lymphopenia CD8+	5 (16)	
Hypogammaglobulinemia (<3 SD according to age)	3 (9.7)	
No abnormality	17 (54)	
No data	6	

A majority of patients were diagnosed during their first year of life (22/37), with five patients diagnosed during their first 3 months of life. The proportion of cases with MI decreased with age, ranging from 7% in the first year of life, 4% in the second, 2% in the third, to less than 1% at >5 years. At the time of MI diagnosis, all patients had multi-system LCH, with 26 (70%) having at least one risk organ involved (liver/spleen or hematological dysfunction); lung involvement was excluded from the definition of risk organ.

MI was found at the first occurrence of the disease in 34 cases, and at the onset of reactivation in three. The initial symptoms leading to LCH diagnosis were related to MI in 13 cases (35%); in the other cases, MI was discovered in the work-up of previously diagnosed LCH. The other initial sites of the disease were skin (28 cases, 75%), bone (21 cases, 56%), lung (15 cases, 40%), hematopoietic system (17 cases, 46%), and liver (16 cases, 43%). The disease scores ranged from 2 to 16 (median score, 5), with only 11 patients categorized as high risk (score > 6) and a majority of patients (N = 24) being of intermediate risk (score of 3–6). Twenty-three patients had general symptoms, that is, fever more than 38.5°C and asthenia with karnofsky indice minor than 80%. Clinical symptoms preceding the observation of MI varied: 10 had only a cough, 4 had respiratory distress, 2 had superior vein cava syndrome, and 1 had tachypnoea without distress. Among the 15 patients with respiratory symptoms, 5 had lung involvement, which may have been partly responsible for these symptoms.

**Fig. 1:**
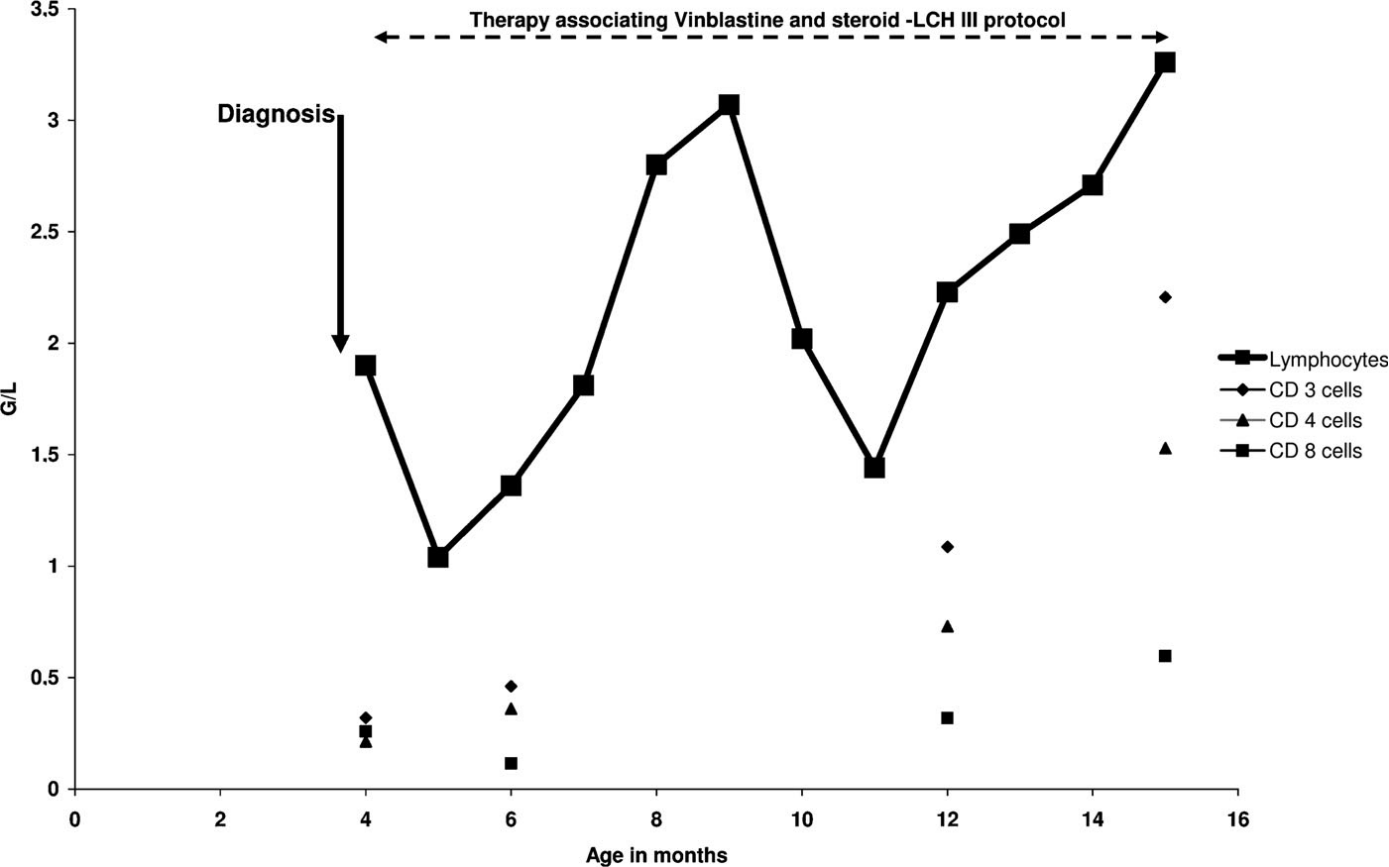
Evolution of lymphocyte count and CD3, CD4, and CD8 subsets at diagnosis, during therapy, and at the completion of the therapy in patient UPN 1506627.

**Fig. 2:**
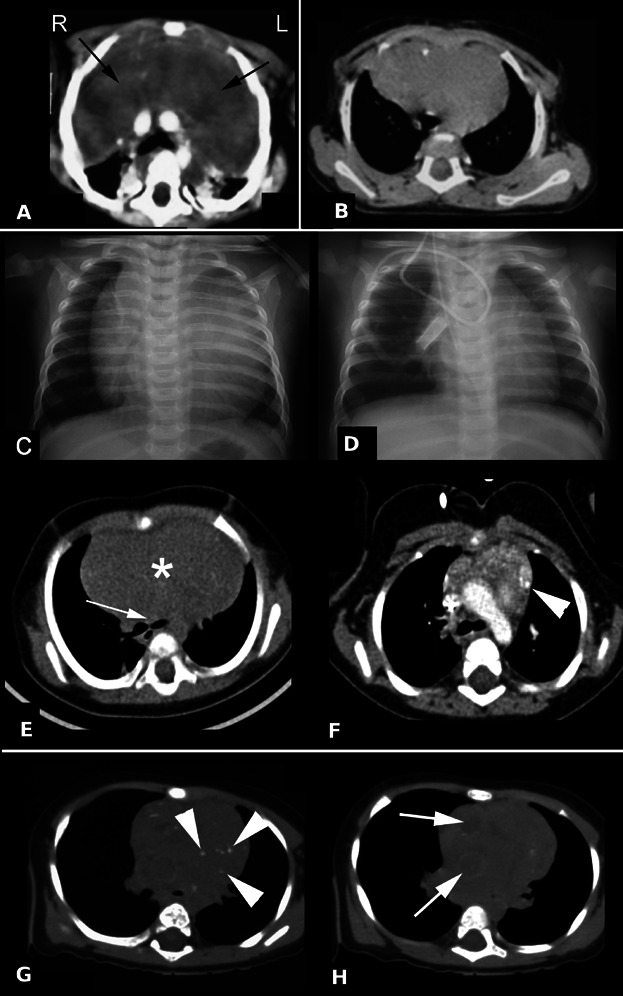
**A**,**B**: Thoracic CT of patient UPN 2106425: an 8-month-old female with respiratory distress at diagnosis. A: Huge enlargement of the thymus (black arrows). B: Evolution after a 6-week course of vinblastine and steroid; CT demonstrates decreased thymus involvement. **C**–**F**: Patient UPN 1506627, seen at 4 months of age for a skin rash. C: Chest X-rays with thymus enlargement. D: Chest X-rays after a 6-week course of vinblastine, showing normalization of thymus size. E: CT scan at time of initial diagnosis with thymus enlargement (*) and tracheal pushback (white arrow). F: Evolution after a 6-week course of vinblastine and steroid, demonstrating the decreased thymus size, but calcifications are now present (arrowhead). **G**,**H**: Thoracic CT of patient UPN 1506102. G: Presence of multiple calcifications (arrowheads). H: Coexistence of intra-thymic cysts (white arrows).

At diagnosis, six patients had lymphopenia with deficiency of CD4 and CD8 T lymphocytes, which was profound in two cases among the 14 patients assessable for these criteria. Figure 1 depicts the lymphocyte status of patient UPN 1506627, both at diagnosis and during therapy; this patient's iconography is reported in Figure 2A,B. One child had hypogammaglobulinemia. In the other patients, the immunological control was normal or not checked.

### Radiological Presentation

All patients had chest X-rays at diagnosis, and 32 also had thoracic CT performed. Tomography emission positron computed tomography (PET-CT) was performed in two cases. Table III presents an overview of the CT features at diagnosis. Although the anatomic delimitation was quite difficult, it was found that 14 patients had only thymic involvement, 10 had only mediastinal lymph node involvement, and 13 had combined (thymus and lymph node) involvement. Sixteen patients presented punctuate calcifications in the thymus, which were visible on CT (Fig. 2F,G, arrowheads) but not on chest radiography. Other signs included a heterogeneous thymus in 18 patients, two of which also exhibited cysts (Fig. 2H, white arrows) besides the calcifications (Fig. 2G, arrowheads). Twelve patients had a nodular thymus with contour modifications, referred to as a polycyclic thymus. Tracheal compression was reported in five patients, tracheal deviation in five, cava vein thrombosis in two, osseous erosion in four (two cases of sternum and two of vertebral erosion), and pericardic effusion in three cases. Abnormalities of the parenchymal lung (cyst or nodules) were found in six patients simultaneously with MI. MI may be analyzed as an involvement of the thymus, the mediastinal nodes or both. Such differences may be difficult to analyze on CT scan. However, if we considered our observation, patients with mediastinal nodes only were older (1.8 years) than patients with thymus only (0.8 years) and patients with thymus and nodes (1 year; *P* = 0.015) while anatomic extension of the disease was about the same between the three subgroups, except the group of patients with thymus involvement which exhibited less bone involvement (41%) compare to the two other groups (80%).

**Table III:** Summary of CT Features at Diagnosis

	Number of patients	%
Mediastinal involvement		
Heterogeneous thymus	18	49
Polycyclic thymus	12	32
Thymic cysts	2	5
Punctuate calcifications	16	43
Mediastinal lymph nodes	17	46
Posterior mediastinal infiltration	4	11
Effects of mediastinal involvement		
Tracheal compression	5	13.5
Tracheal pushback	5	13.5
Pericardic effusion	3	8
Vena cava thrombosis	2	5
Sternal erosion	2	5
Vertebral erosion	2	5

### Pathological Findings

LCH was histologically confirmed in all patients by demonstrating CD1a-positive cells in biopsies from bone, skin, and lymph nodes. Thymic involvement was also histologically confirmed in four patients, and mediastinal lymph nodes were biopsied in one patient. The pathological cells exhibited an eosinophilic appearance of the cytoplasm and few mitotic figures. Immunohistochemical analysis revealed strong positivity of these cells for CD1a and S-100 protein. *BRAF* p.V600E mutations were only studied in two patients, and were detected in both cases, including the tumor DNA obtained from one thymic sample.

### Characteristics of Patients With Mediastinal Involvement Compared With the Overall Patient Sample

Some demographic features and organ involvements differed between the 37 patients with MI and the 1,386 patients without MI in the registry. Median age at diagnosis was 0.75 years in the MI group versus 3.2 years in the non-MI group (*P* < 0.001). Gender did not differ between the groups. At the maximal extent of the disease, significant differences (with *P* < 0.001 or below) were observed between the MI group versus the non-MI group with regards to the prevalence of the organ involvement, with less bone involvement (65% vs. 82%), and more involvement of skin (78% vs. 33%), lung (49% vs. 9%), hematopoetic dysfunction (49% vs. 9%), liver (46% vs. 9.5%), and spleen (32% vs. 8%). Central nervous system and pituitary involvement did not statistically differ between the two groups. The natural history of these patients was marked by a higher rate of reactivation (48% vs. 33%; *P* = 0.01) and a higher mortality rate (16% vs. 4%; *P* = 0.01) compare to non-MI group.

### Therapy and Outcome

**Fig. 3:**
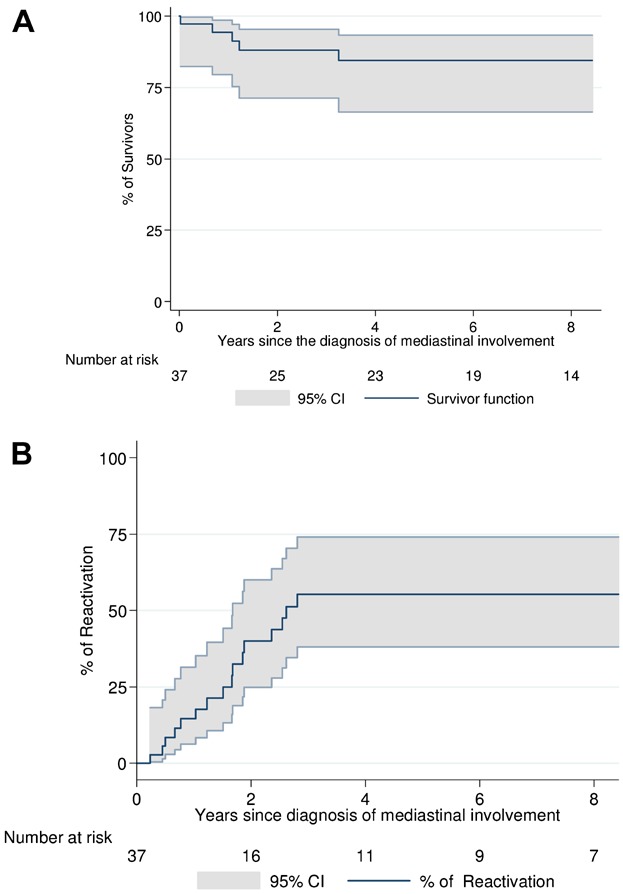
**A**: Overall survival and 95% CI. **B**: Risk of reactivation since diagnosis and 95% confidence interval (CI). In case of death before reactivation, the data are censored at the time of death.

Of the 34 patients with MI present at the initial LCH diagnosis, 32 were treated with corticosteroids (prednisone) at a dose of 40 mg/m^2^/day and vinblastine (6 mg/m^2^/day), sometimes in association to additional drugs. One patient died before any therapy and one patient was treated with steroid alone. If the patients were included in therapeutic LCH II and LCH III trials, VP16 (N = 2) or methotrexate (N = 4) was administered according to the randomization of the trial. The three patients with MI at relapse were each treated at their initial LCH diagnosis with the same doses of prednisone and vinblastine. At relapse, one patient was treated with vinblastine and prednisone, one with 2-chlorodeoxyadenosine (2-CdA) alone, and one received VP16, cytarabine, and cyclosporine. After 6 weeks of treatment, according to the evaluation criteria of the Histiocyte Society, the disease status was considered as non-active in four patients (11%), active disease better in 29 (78%), including one who had received only steroid treatment, active disease—stable in three (8%) and lastly active disease—worse in one patient (2.7%) who died from the disease. The disease activity score decreased for all patients, except those who died from the disease, however it remained above five in six patients, which can be considered a poor response. All treated patients presented with decreased volume of MI; 15 patients had a residual mass after 6 weeks. Even in cases with poor response to the initial therapy, MI was not considered to be life threatening, or even as a therapeutic target in the latter course of therapy. Sixteen patients experienced at least one disease relapse and the 5-year relapse rate was only 45% (95% CI, 26–62%; Fig. 3B). Three of these relapses involved the thymus in patients who did not have MI at initial diagnosis, at 1 or 3 years after the initial diagnosis. Treatment of refractory disease or relapse was managed according to national guidelines, when available. Four patients received 2-CdA and cytarabine as a second-line therapy, and four patients received monotherapy with 2-CdA.

The overall survival (Fig. 3A) was 87% (95% CI, 69–95%). Five patients died. One of them was the youngest child of the cohort, a 15-day-old newborn; a few days after diagnosis and before receiving any therapy, this patient experienced multi-organ failure and sudden death, possibly due to tracheal compression. Another patient died during the course of the disease due to a parenteral nutrition overinfusion. One patient, whose disease was initially controlled, died during a relapse of the disease involving risk organs that was being treated with vinblastine, steroid, and procarbazine; the cause of death was respiratory distress, related to the disease itself. Two patients with multivisceral refractory disease were treated with 2-CdA and cytarabine, and died from sepsis. Among the 35 patients treated with systemic therapy, severe infection was observed in seven patients during the first course of systemic therapy. One patient presented both cytomegalovirus and *Pneumocystis jirovecii* infections, and six patients presented septicemia (*Staphylococcus aureus* in three cases, and one case each of group B *streptococcus*, group A *streptococcus*, and *Pseudomonas aeruginosa*). This incidence of severe infection was unusually high among patients receiving the standard vinblastine and steroid courses; however, immunological parameters were not abnormal among the patients with severe infection. Six patients presented lymphopenia and/or hypogammaglobulinemia in the initial work-up, but these patients experienced no severe infection, and their lymphocytes counts and immunoglobulin levels normalized a few months after beginning histiocytosis treatment.

## DISCUSSION

Langerhans histiocytosis with thymic and/or mediastinal localization is rarely reported and probably under-diagnosed. Review of the literature from 1963 to 2010 identified 50 described cases, almost always reported as single case reports 5,6,8,9,11–16,19–21,23–36 and infrequently as small patient surveys (including four, four, four, five, and three cases, respectively) 7,10,17,18,22. In the present study, we investigated a large cohort of pediatric patients with LCH and estimated that mediastinal and/or thymic involvement occurs in up to 2.6%. This estimation is based on all cases included in the national French registry since 1983. The proportion was found to be largely dependent on age, ranging from about 7% in infants less than 1 year of age, and decreasing with age to less than 1% in older children. It is noteworthy the total median age of previously reported cases (Table I) was 1.55 years; the literature included six adults older than 18 years 31–36.

The radiological findings in our cohort confirmed the previously reported morphological description and provide greater insight into the natural history of thymic involvement. A majority of patients had an anterior mediastinal or thymic enlargement 18. CT scan revealed calcification in about half of our patients as previously described 13,16,21, which was not seen on standard chest X-rays. Of note, we found these calcifications exclusively in the thymus as has also been previously reported 9 but not in the mediastinal lymph nodes. Another sign of disease involvement was the presence aeric cysts in the thymus, described in children 10 and in adults 35,36. We also found a notable discrepancy between the radiological description and the clinical symptoms; only a third of cases presented symptoms related to MI, while two-thirds had no local symptoms despite a huge volume of thymus involvement.

In addition to providing a description of MI, our study found that such involvement was always associated with multi-systemic diseases, with half of the patients having affected risk organs. This is consistent with the majority of cases reported in the literature (Table I), although some cases have been reported as exclusively localized to the thymus 21,23. The distribution of the different organ involvement is different in patients with MI, compare to the common presentation of LCH in children 4. Patients with MI had less frequently bone lesions and more frequently skin involvement contrary to patients without MI. This difference is probably associated to the younger age of the majority of our patients; the median age of our patients was 0.7 years versus 3.5 years for all children with LCH 4. Consequently to a more aggressive and extended disease, systemic therapy was prescribed in almost all of the patients with MI. The proportion of refractory patients seems in agreement with what has been commonly observed in patients with risk organ involvement 39.

Although thymic involvement may result in significant adverse symptoms, it is not considered as a major organ determining the prognosis. Four out of the five deaths observed in this survey were not related to MI, but rather to the already known risk organs, the toxicity of second-line therapy approaches (like 2-CdA with cytarabine), or supportive care (parenteral nutrition). However, although the overall prognosis was not related to the MI, we were surprised by the high rate of severe infections; 7 out of 37 patients is far higher than the rate commonly observed with the standard vinblastine and steroid courses 40. This rate of infections is closer to the rate observed during induction of acute lymphoblastic leukemia 41. This infection rate may be partially explained by the young age of the patients, as well as by the alteration of immunocompetence by the thymus involvement, as transiently observed in six cases. Because such patients had several infection risk factors at the same time such as young age, central line, skin effraction, thymus disease, and chemotherapy and they must probably have to be considered as being at a high risk of infection.

## CONCLUSIONS

MI is rare in Langerhans cell histiocytosis, except in young patients. Although frequently asymptomatic, such involvement may be life threatening and is more commonly associated with risk organ involvement. Diagnosis of MI could be difficult with plain X-rays, and CT is very useful in this cases. Thymic calcification on CT is the most typical lesion found. The possible association with immunodeficiency and the more frequent occurrence of severe infection must be noted, and such patients should be considered to be at a high risk of infections during their initial therapy.
